# Intraspecific Variation in *Pinus Pinaster* PSII Photochemical Efficiency in Response to Winter Stress and Freezing Temperatures

**DOI:** 10.1371/journal.pone.0028772

**Published:** 2011-12-29

**Authors:** Leyre Corcuera, Eustaquio Gil-Pelegrin, Eduardo Notivol

**Affiliations:** Forest Resources Unit, Research and Technology Agricultural Centre (CITA), Government of Aragon, Zaragoza, Spain; University of California – Davis, United States of America

## Abstract

As part of a program to select maritime pine (*Pinus pinaster* Ait.) genotypes for resistance to low winter temperatures, we examined variation in photosystem II activity by chlorophyll fluorescence. Populations and families within populations from contrasting climates were tested during two consecutive winters through two progeny trials, one located at a continental and xeric site and one at a mesic site with Atlantic influence. We also obtained the LT_50_, or the temperature that causes 50% damage, by controlled freezing and the subsequent analysis of chlorophyll fluorescence in needles and stems that were collected from populations at the continental trial site.

*P. pinaster* showed sensitivity to winter stress at the continental site, during the colder winter. The combination of low temperatures, high solar irradiation and low precipitation caused sustained decreases in maximal photochemical efficiency (*F_v_*/*F_m_*), quantum yield of non-cyclic electron transport (Φ_PSII_) and photochemical quenching (*qP*). The variation in photochemical parameters was larger among families than among populations, and population differences appeared only under the harshest conditions at the continental site. As expected, the environmental effects (*winter* and *site*) on the photochemical parameters were much larger than the genotypic effects (*population* or *family*). LT_50_ was closely related to the minimum winter temperatures of the population's range. The dark-adapted *F_v_*/*F_m_* ratio discriminated clearly between interior and coastal populations.

In conclusion, variations in *F_v_*/*F_m_*, Φ_PSII_, *qP* and non-photochemical quenching (*NPQ*) in response to winter stress were primarily due to the differences between the winter conditions and the sites and secondarily due to the differences among families and their interactions with the environment. Populations from continental climates showed higher frost tolerance (LT_50_) than coastal populations that typically experience mild winters. Therefore, LT_50_, as estimated by *F_v_*/*F_m_*, is a reliable indicator of frost tolerance among *P. pinaster* populations.

## Introduction

*Pinus pinaster* progeny trials have been established throughout Spain for conservation purposes and to analyze the growth and physiological adaptations of different seed sources to several soil and meteorological conditions [1]. These trials were designed with a nested structure to examine families within populations. The term “family” refers to a group of individuals that have one or both parents in common (half-sib and full-sib families, respectively). “Population” denotes the group of individuals within which there is gene exchange, and “provenance” refers to the geographic origin of the population. Natural populations have been subjected to selection by their particular set of local environmental conditions and may differ in performance when grown at a common site. Progeny trials are the best way to evaluate the genetic value of selected parents to determine the population best suited to particular climatic conditions. The relationships among the traits that are related to yield (survival, wood volume) and wood quality (polycyclism) have allowed the selection of populations that are well suited to the environmental conditions at several trial sites [2]. In particular, the populations and families evaluated here have shown differentiation and plasticity in growth and physiological parameters related to drought, such as carbon isotope composition [3], vulnerability to xylem embolism [4] and accumulation of phytoregulators [5].

As in other evergreen species, the leaves of the maritime pine allow CO_2_ uptake during the whole year, whenever favorable climate conditions occur. Physiological parameters related to CO_2_ fixation, such as the rate of photosynthesis and the activity of photosystem II (PSII), decline with abiotic stress [6]. Drought stress is the main factor that restrains growth and survival in the Mediterranean climate. In humid and cold forest zones, low temperatures are the limiting factor. However, frost-induced reductions in photosynthesis are reversible. The recovery time after frost varies with species, frost intensity, cold hardiness [7] and the light exposure of the needles [8]. Natural populations experience several simultaneous environmental stresses that have interactive effects. The combination of low winter temperatures and high solar radiation increases the amount of absorbed light that cannot be used by the plant, leading to greater reductions in PSII photochemical efficiency in conifers [9] and broadleaf evergreens [10]. In Mediterranean areas with a continental climate (large seasonal temperature differences: hot summers and cold winters), cold hardiness and frost resistance of evergreens are a matter of concern [11]. In this case, winter stress can be more harmful for the photosynthetic apparatus than summer stress [12] as low winter temperatures coincide with high solar irradiation and drought stress. However, the photoprotection processes, such as the increase of non-photochemical quenching (*NPQ*), ensure the dissipation of excess light and prevent chronic photoinhibition, allowing the recovery of photosynthesis and PSII photochemical efficiency when temperatures increase in the spring [13].

Chlorophyll fluorescence has been a useful tool for allowing plant physiologists to detect the effects of biotic and abiotic stresses on light-processing physiology [14]. Changes in fluorescence parameters in response to environmental stresses, such as low [e.g., 15] and high temperatures [e.g., 16], high light intensity [e.g., 17] and drought [e.g., 18], have been widely documented at the species level. Intra-specific variation in the tolerance to low temperatures among populations is essential for seed transfer between geographic regions during reforestation [19], [20]. A positive relationship between the latitude of provenance and *F_v_/F_m_* ratio after frost events was observed in *Pinus contorta* and *Pinus sylvestris* populations [21]. At the family level, Koeehn et al. [22] found genetic variation in *qP* and *F_v_/F_m_* and a positive relationship between *qP* and growth in *Pinus elliottii*.

Chlorophyll fluorescence is also an accurate indicator of freezing injury. Freeze tolerance, or the ability of plants to survive subfreezing temperatures, can be measured by the dark-adapted *F_v_/F_m_* ratio. However, chlorophyll fluorescence as a screening method for freezing injury in conifers has been investigated mainly at the species level. It has been shown as a fast and reliable technique for establishing the freeze temperature that causes 50% damage (LT_50_) in *Pinus sylvestris* needles and stems (cortical bark chlorenchyma) [23]. The estimation of the LT_50_ of different genotypes by the *F_v_/F_m_* ratio has been investigated mainly in crop plants [e.g., 24]. Few studies have measured intraspecific variation in LT_50_ [25] and cold tolerance [26] by means of chlorophyll fluorescence in forest tree species.

To date, only the short-term and seasonal responses of photosynthesis to temperature have been documented in *P. pinaster* populations [27]. Meanwhile, to our knowledge, no studies have investigated the effect of winter stresses (high irradiation, low temperatures and drought) or the temperature that causes freezing injury by means of chlorophyll fluorescence at the species, population or family level in *P. pinaster*. In this work, we tested several *Pinus pinaster* genotypes with different field survival rates in provenance-progeny trials during two consecutive winters at two sites that vary in productivity due to contrasting altitudes and precipitation regimes. One site is at a low elevation near the Atlantic and is exposed to wet and mild winters, and the other site is at an interior mid-high elevation with colder and drier winters. Our objectives were (1) to assess the environmental and genetic variability in PSII photochemical performance in response to winter stress (by means of Φ*_PSII_*, *F_v_/F_m_*, *q_P_* and *NPQ*) and (2) to determine whether the LT_50_ (estimated by *F_v_/F_m_*) was related to the original climate of the populations. Our results indicate that there is genetic variation in photochemistry in response to winter stress, mainly at a family level. We present evidence that population differences in the freezing tolerance of needles and stems are consistent with the winter temperatures of the climate of origin, providing evidence in support of local adaptation.

## Results

### The effect of climate on photochemical efficiency and survival

**Figure 1:**
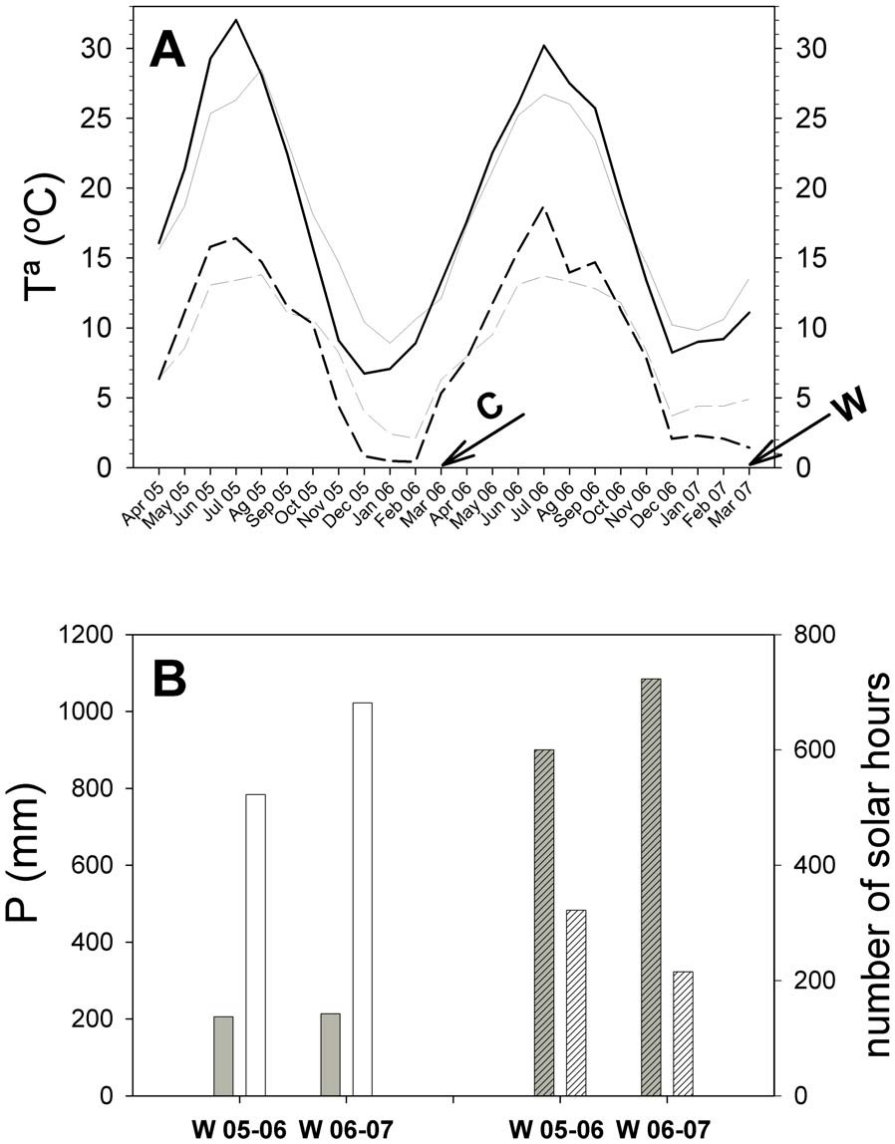
Climatic data. (A) Monthly mean of the maximum temperatures in *XE* (thick line) and *ME* (hairline) and of the minimum temperatures in *XE* (dashed line) and *ME* (dashed hairline). Arrows indicate the measurement dates. C: cold winter, W: warm winter. (B) Sum of the precipitation in autumn and winter in *XE* (grey bars) and *ME* (white bars). Sum of the solar hours in *XE* (gray hatched bars) and *ME* (white hatched bars). W 05–06: winter of 2005–2006, cold winter. W 06–07: winter of 2006–2007, warm winter.

**Figure 2:**
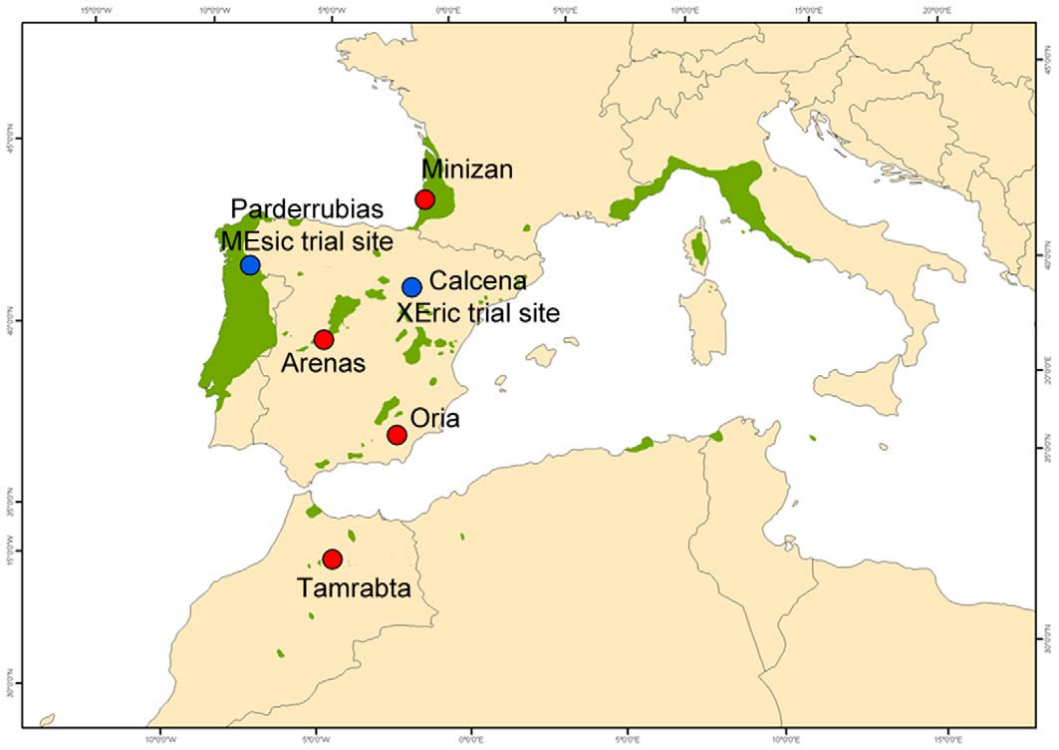
Locations of the populations and trial sites.

The monthly means of the minimum autumn and winter temperatures were lower in 2005–2006 than in 2006–2007 at both sites (Fig. 1A). The study was conducted during the late winter of 2006 (cold winter) and 2007 (warm winter, Fig. 1A). Two progeny trials were conducted (Fig. 2). The total autumn and winter precipitation at the mesic site (ME) was four to five times higher than that at the xeric site (*XE*). The cumulative radiation at the *XE* was close to two (winter 2005–2006) or three times higher (winter 2006–2007, Fig. 1B) than that at the *ME*.

The first component of the principal component analysis (PCA) absorbed 99% of the total variation. The main variables were the following, in order of relevance: continentality (extreme winter and summer temperatures), annual precipitation, precipitation during the wettest quarter and precipitation during the coldest quarter. Populations were arranged according to the bioclimatic index (Table 1): *Arenas* and *Tamrabta*, the populations from continental climates, followed by *Oria* from south Spain, are subjected to more stressful conditions at their sites of origin, similar to those experienced at the xeric site. The *Mimizan* population, from France, is native to a climate similar to that at the mesic site.

**Table 1:** Location and climatic data for the provenance-progeny trials and seed sources.

Locality	Calcena (XE)	*Arenas*	*Tamrabta*	*Oria*	Parderrubias (*ME*)	*Mimizan*
**Bioclimatic Index**	−794	−778	−613	−587	841	1026
**Latitude (N)**	41°37′	40°30′	33°71′	37°30′	42°14′	44°8′
**Longitude (W)**	1°44′	4°24′	4°74′	2°20′	7°56′	1°10′
**Elevation (m)**	1017	1359	1600	1232	460	0–80
**P (mm)**	461	692	950	351.5	722	1202
**T (°C)**	12.3	14.6	13.1	14.4	14.4	13.0
**TM (°C)**	28.6	34.2	33.0	30.0	27.8	25.0
**Tm (°C)**	1.1	0.3	−0.9	3.0	1.5	2.0

Populations and sites are arranged according to the Bioclimatic Index (lower values indicate drier continental climates). P: mean annual precipitation (mm); T: mean annual temperature (°C); TM: mean of the maxima in the month with the highest mean temperatures (°C); Tm: mean of the minima in the month with the lowest mean temperatures (°C); *XE*: continental and xeric site; *ME*: mesic site.

Despite the low autumn and winter precipitation at the *XE*, no trees at any of the trial sites had signs of water stress. Predawn water potential was lower after the cold winter (*ME*: −0.03±0.01 MPa in the cold winter; −0.01±0.01 MPa in the warm winter; *XE*: −0.87±0.03 MPa in the cold winter; −0.58±0.03, MPa in the warm winter).

As expected, environmental conditions (*winter*, *site*, and their interaction, *site x winter*) had a major influence on photochemical parameters (Table 2). All three populations were sensitive to the combination of winter stresses during the cold winter at the continental site, which was characterized by low temperatures, low precipitation (approximately 200 mm in autumn and winter) and high irradiation (PAR above 2000 µmol photons m^−2^ s^−1^).

**Table 2:** Summary of ANOVA significances.

			*F_v_*/*F_m_*		Φ_PSII_		*qP*		*NPQ*	
Source of variation	Num *DF*	Den *DF*	*F*-Value	Pr>*F*	*F*-Value	Pr>*F*	*F*-Value	Pr>*F*	*F*-Value	Pr>*F*
***Winter***	2	541	111.96	**<0.0001**	101.11	**<0.0001**	185.55	**<0.0001**	45.10	**<0.0001**
***Site***	1	541	10.84	**0.0011**	2.45	0.1184	4.44	**0.0355**	3.47	**0.0630**
***Winter*** ** x ** ***site***	2	541	38.73	**<0.0001**	0.53	0.5906	3.81	**0.0227**	3.42	0.0334
***Pop***	2	541	0.55	0.5772	0.27	0.7657	1.03	0.3595	1.81	0.1654
***Pop*** ** x ** ***winter***	4	541	1.02	0.3975	1.79	0.1284	1.55	0.1851	2.46	**0.0443**
***Pop*** ** x ** ***site***	2	541	1.13	0.3225	1.56	0.2115	2.06	0.1290	6.11	**0.0024**
***Pop*** ** (** ***winter*** ** x ** ***site*** **)**	4	541	1.72	0.1448	3.28	**0.0114**	1.23	0.2968	1.02	0.3982
***Fam*** ** (** ***Pop*** **)**	42	541	1.33	0.0846	2.10	**0.0001**	4.10	**<0.0001**	1.58	**0.0128**
***Fam*** ** (** ***Pop*** ** x ** ***winter*** **)**	7	541	1.14	0.2028	1.77	**0.0002**	2.17	**<0.0001**	1.76	**0.0002**
***Fam*** ** (** ***Pop*** ** x ** ***site*** **)**	75	541	1.82	0.0807	1.34	0.2287	3.31	**0.0019**	3.15	**0.0029**
***Fam*** ** (** ***Pop*** ** x ** ***winter*** ** x ** ***site*** **)**	12	541	1.29	0.2176	1.78	**0.0486**	2.76	**0.0012**	2.08	**0.0169**

*F*_v_/*F*_m_: maximum potential PSII efficiency. Φ_PSII_: actual PSII efficiency. *qP*: photochemical quenching. *NPQ*: non-photochemical quenching. *Winter*: studied period (winter 2005–06, winter 2006–07). *Site*: location of the provenance-progeny trials. Num *DF*: number of degrees of freedom. Den *DF*: Denominator of degrees of freedom. Pr: Probability. *F*: F-values. Bold numbers denote values for which Pr>0.05.

**Figure 3:**
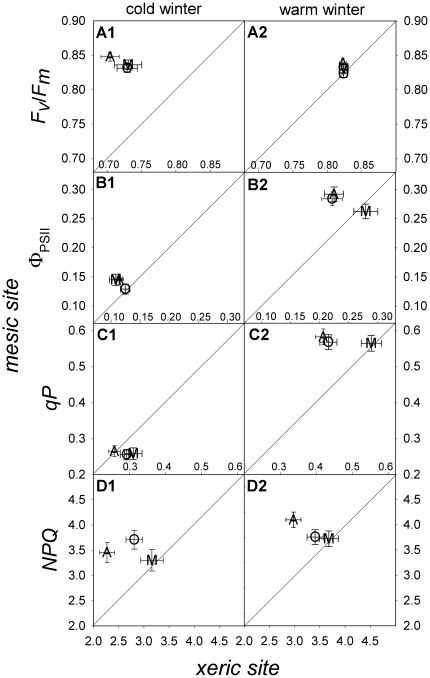
Population variation in the photochemical parameters by site and winter. Bi-directional least squared means ± standard errors of the photochemical parameters for the populations (A: *Arenas*, O: *Oria*, M: *Mimizan*) present in the xeric (axis X) and mesic (axis Y) trial sites. Maximum potential (*F*_v_/*F*_m_) and actual (**Φ**_PSII_) PSII efficiency and photochemical quenching (*qP*) and non-photochemical quenching (*NPQ*) after the cold (A1, B1, C1, D1) and warm winter (A2, B2, C2, D2) are shown. Diagonal lines indicate equal values at both sites. Values shown represent the mean of the values from 6 to 10 families from 3 populations.

After the cold winter, there was a reduction in *F_v_*/*F_m_* values, i.e., a down-regulation of PSII efficiency due to a cumulative effect of winter stresses at the *XE* (*F_v_*/*F_m_*: 0.70–0.73±0.01 compared to 0.83–0.85±0.006 at the *ME*, Fig. 3A1). *F_v_/F_m_* values were within [28] or below [29] the ranges described in previous studies of *Pinus halepensis* and did not decrease significantly, indicating that the remaining PSII reaction centers were photochemically active during the winter.

The proportion of energy used for photochemistry (i.e., the actual photochemical efficiency, Φ_PSII_) and the proportion of PSII open centers, *qP*, had similar behavior at both sites and during both winters (Figs. 3B1, 3C1, 3B2 and 3C2). Both Φ_PSII_ and *qP* exhibited maximum values after the mild winter and minimum values after the cold winter at both sites.

At the xeric trial, the French population (*Mimizan*) was more sensitive to drought than any other population. In 2005, survival rates were 48%, 50% and 40% for the *Arenas*, *Oria* and *Mimizan* populations, respectively. In 2006, survival rates were 35%, 36% and 23% for the *Arenas*, *Oria* and *Mimizan* populations, respectively. High mortality was observed during both years due to severe drought and degraded soils. In the mesic trial, survival was close to 100%, and the posterior mortality (<5%) was the consequence of human error, which occurred when several of the trees were cut with farming implements while performing mechanical weed control.

### Intraspecific winter variation in PSII photochemical parameters

Differences among populations were only observed at the *XE* (Table 3), and some significant heritabilities were found at the *XE* as well (Table 4). Population differences in Φ_PSII_, *qP* and *NPQ* after the warm winter (Table 3; Figs. 3B2, 3C2, 3D2) and in *NPQ* after the cold winter were observed (Table 3; Fig. 3D1). The *Mimizan* population showed a significantly higher Φ_PSII_ and *qP* after the warm winter than the *Arenas* and *Oria* populations (Fig. 3B2, 3C2; Table 3). Heat dissipation was lower in the *Arenas* population, which inhabits a cold climate, after both winters (Figs. 3D1, 3D2; Table 3).

**Table 3:** Summary of ANOVA significances by *site* and *winter*.

					*F_v_*/*F_m_*		Φ_PSII_		*qP*		*NPQ*	
*Winter*	*Site*	Effect	Num *DF*	Den *DF*	*F*-Value	Pr>*F*	*F*-Value	Pr>*F*	*F*-Value	Pr>*F*	*F*-Value	Pr>*F*
**Cold**	***XE***	***Pop***	2	91	1.02	0.3632	0.83	0.4395	1.91	0.1547	6.47	**0.0024**
**Cold**	***XE***	***Fam (Pop)***	21	91	1.20	0.2694	2.45	**0.0018**	1.95	**0.0159**	1.88	**0.0220**
**Cold**	***ME***	***Pop***	2	62	1.80	0.1736	1.12	0.3320	0.11	0.8978	1.09	0.3433
**Cold**	***ME***	***Fam (Pop)***	19	62	1.98	**0.0229**	1.74	**0.0532**	1.31	0.2804	1.51	0.1142
**Warm**	***XE***	***Pop***	2	98	0.01	0.9895	2.77	0.0677	7.80	**0.0007**	4.66	**0.0117**
**Warm**	***XE***	***Fam (Pop)***	23	98	1.98	**0.0113**	2.13	**0.0056**	3.67	**<0.0001**	1.72	**0.0358**
**Warm**	***ME***	***Pop***	2	98	1.98	0.1442	1.50	0.2286	0.19	0.8265	1.85	0.1633
**Warm**	***ME***	***Fam (Pop)***	26	98	1.20	0.2562	2.59	**0.0004**	5.73	**<0.0001**	1.76	**0.0251**

Abbreviations are the same as those in Table 2.

**Table 4:** Population heritabilities (*h^2^*) with non-zero significance, by *site* and *winter*.

Winter	*Site*	*F*_v_/*F*_m_	Φ_PSII_	*qP*	*NPQ*
**Cold**	***XE***	0	0	0	**0.39**
**Cold**	***ME***	0	0	0	0
**Warm**	***XE***	0	0.03	**0.41**	**0.23**
**Warm**	***ME***	0.06	0	0	0

Abbreviations are the same as those in Table 2.

**Figure 4:**
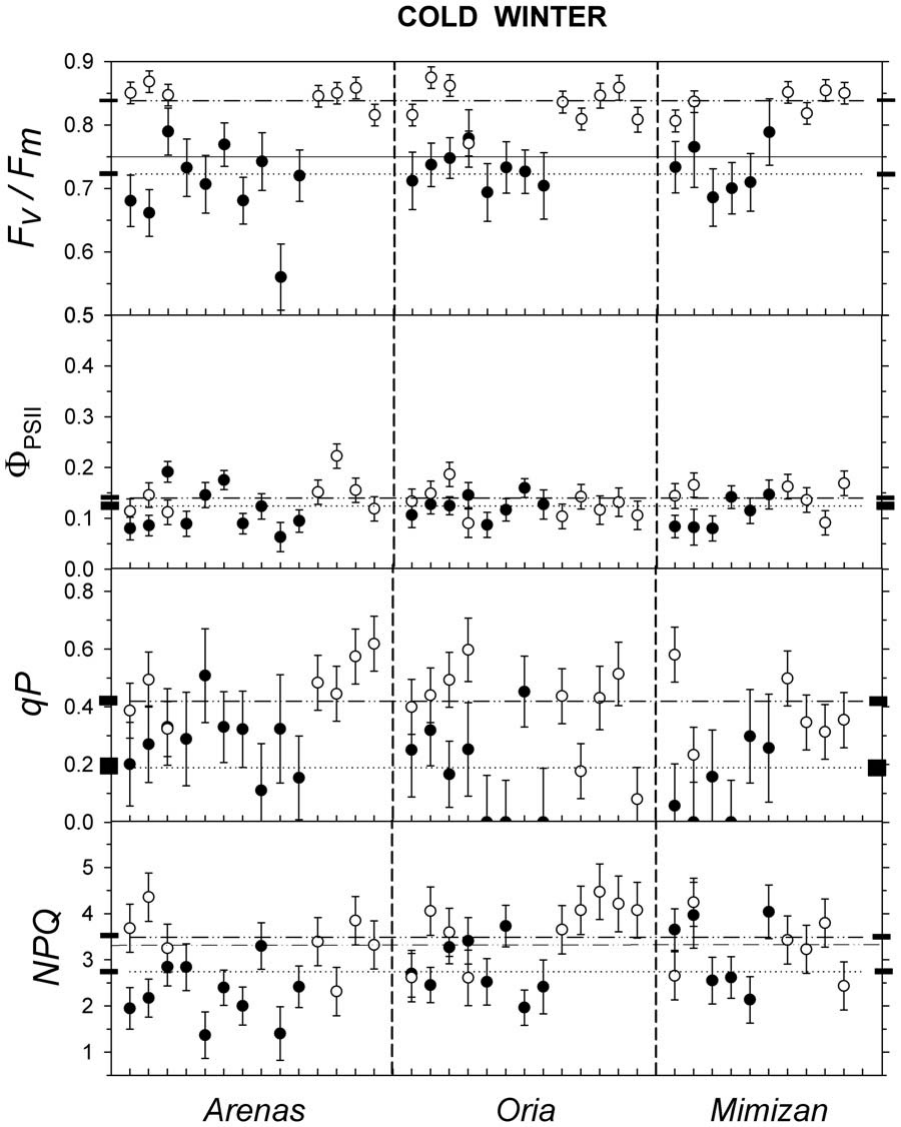
Family variation in the photochemical parameters during the cold winter. Least squared means ± standard errors of the photochemical parameters for families from the *Arenas*, *Oria* and *Mimizan* populations present in the xeric (black circles) and mesic (white circles) trial sites in the cold winter. Families present at both sites are represented at the same position on the X axis. Abbreviations are the same as those in Fig. 3.

**Figure 5:**
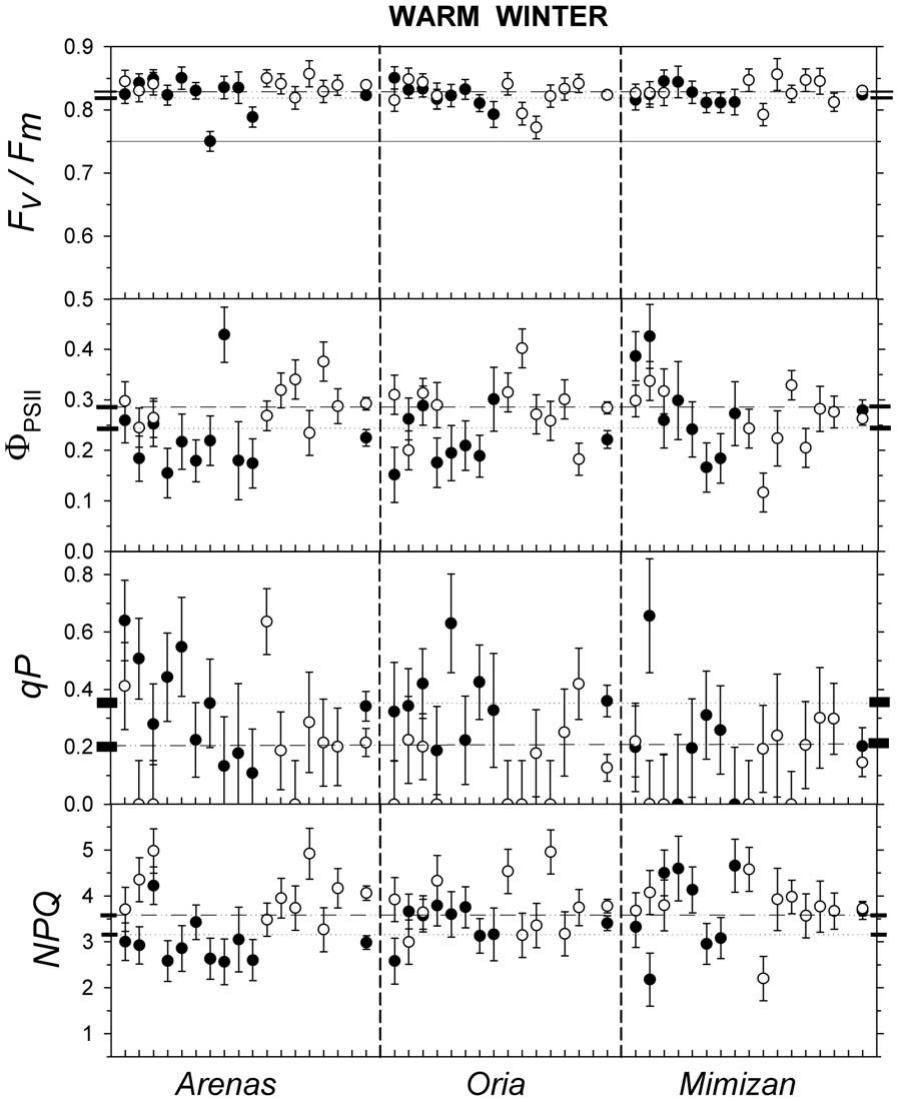
Family variation in photochemical parameters during the warm winter. Least squared means ± standard errors of the photochemical parameters for the families from the *Arenas*, *Oria* and *Mimizan* populations present in the xeric (black circles) and mesic (white circles) trial sites in the warm winter. Families present at both sites are represented at the same position on the X axis. Abbreviations are the same as those in Fig. 3. The Y-axis scale is the same as that in Fig. 4 to highlight the differences between the winters.

The variation in photochemical parameters among families is shown in Figs. 4 and 5. Differences among the families were significant for Φ_PSII_, *qP* and *NPQ* (Table 2) and were significant for *F_v_*/*F_m_* when the parameters were obtained for each site and winter (Table 3). The family and its interaction with *winter*, *site* and *site x winter* were all significant sources of variance for all of the parameters (Table 2).

Survival rates were not related to the photochemical performance of the populations. At the *XE*, the *Mimizan* population exhibited lower survival rates that were not associated with an inferior photochemical performance.

### Intraspecific variation in LT_50_

**Figure 6:**
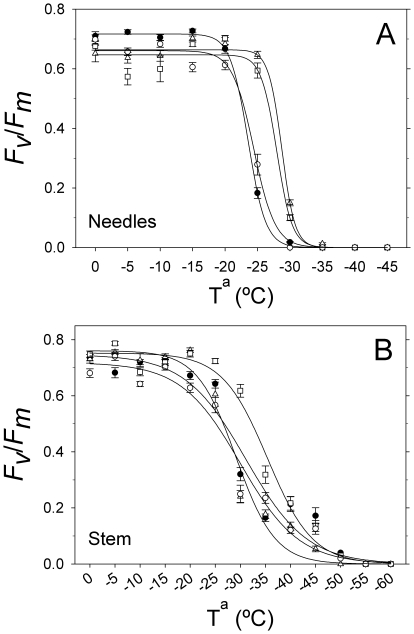
Population variation in the freezing tolerance of needles and stems. The relationship between the temperature (°C) and maximum potential PSII efficiency (*F*_v_/*F*_m_) in the needles (A) and stems (B) of *Pinus pinaster* populations is shown: *Oria* (filled circles), *Mimizan* (open circles), *Tamrabta* (squares) and *Arenas* (triangles). Error bars indicate standard errors of the mean values.

**Figure 7:**
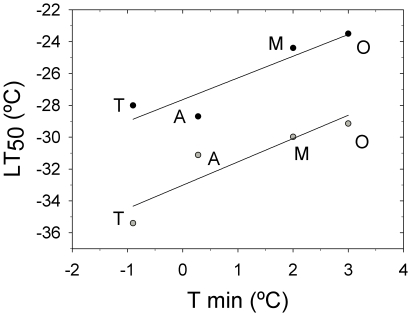
Populations ranked according to LT_50_. The relationship between the LT_50_ of the needles (black circles) or stems (grey circles) and the mean minimum temperature of the coldest month (Tmin) for the population origin is depicted. T: *Tamrabta*, A: *Arenas*, O: *Oria*, M: *Mimizan*.

Figure 6 shows the evolution of *F_v_*/*F_m_* in needles (A) and stems (cortical bark chlorenchyma, B) exposed to frost temperatures. There was a clear differentiation between the interior and coastal populations. Needle tolerance to freezing temperatures was higher in the populations from locations with a continental climate and low winter temperatures, *Arenas* and *Tamrabta* (LT_50_: −28.7±0.40 and −28.0±0.62, respectively, R^2^ = 0.99, P<0.0001; Fig. 6A, Table 1). The populations from locations with mild winters, *Oria* and *Mimizan*, exhibited lower absolute values of LT_50_ (−23.5±0.10 and −24.4±0.31, respectively, R^2^ = 0.99, P<0.0001; Fig. 6A). However, the frost resistance of the cortical bark chlorenchyma was only significantly higher in *Tamrabta* (−35.4±0.76; R^2^ = 0.99, P<0.0001) than in the rest of the populations (*Arenas*: −31.1±1.62, *Oria*: −29.1±0.88 and *Mimizan*: −30.0±1.30; R^2^ = 0.99, P<0.0001; Fig. 6B). Population differences in LT_50_ were correlated with the mean minimum temperature of the coldest month at the seed source (Fig. 7). LT_50_ decreased with decreasing mean minimum temperatures of the seed source for both needles (R^2^ = 0.85, P<0.05) and stems (R^2^ = 0.84, P<0.05). The ranking of populations according to LT_50_ was similar for needles and stems.

### Relationship among the photochemical parameters

High values of *qP* were associated with high values of Φ_PSII_ (Table 5) in general, and when they were separated by *site* and *winter* (Table 6). Similarly, high values of *F_v_*/*F_m_* were correlated with high values of *NPQ* (Table 5), in general and when they were separated by *site* and *winter* (Table 6). *F_v_*/*F_m_* was positively correlated with Φ_PSII_ and *qP* at both sites after the cold winter. At the *XE*, *NPQ* was negatively correlated with Φ_PSII_ after the warm winter and with *qP* after the cold winter (Table 6).

**Table 5:** Correlations among photochemical parameters.

Parameter	Φ_PSII_	*qP*	*NPQ*
*F*_v_/*F*_m_	**0.24** ***	**0.14** ***	**0.49** ***
**Φ**_PSII_	0	**0.87** ***	**−0.09** *
*qP*	0	0	**0.08** *

Abbreviations are the same as those in Table 2. Bold numbers denote significant values. Significance levels:

***P<0.001,

**P<0.01,

*0.01<*P*<0.05.

**Table 6:** Correlations among the photochemical parameters, separated by *winter* and *site*.

		Xeric site			Mesic site		
Winter	Parameter	Φ_PSII_	*qP*	*NPQ*	Φ_PSII_	*qP*	*NPQ*
**Cold**	*F*_v_/*F*_m_	**0.53** ***	**0.46** ***	**0.62** ***	**0.46** ***	**0.43** ***	**0.43** ***
**Cold**	**Φ**_PSII_	1	**0.79** ***	0.08	1	**0.93** ***	−0.08
**Cold**	*qP*	**0.79** ***	1	**−0.25** **	**0.93** ***	1	0.05
**Warm**	*F*_v_/*F*_m_	0.14	0.04	**0.31** ***	0.08	−0.04	**0.32** ***
**Warm**	**Φ**_PSII_	1	**0.85** ***	**−0.27** **	1	**0.77** ***	−0.10
**Warm**	*qP*	**0.85** ***	1	0.11	**0.77** ***	1	**−0.24** **

Abbreviations are the same as those in Table 2. Bold numbers denote significant values. Signification level:

***P<0.001,

**P<0.01,

*0.01<*P*<0.05.

## Discussion

### The effect of winter stress on PSII photochemical parameters

Environmental factors (*site*, *winter*, *site x winter*) contributed to the phenotypic variation in photochemical parameters to a greater degree than did genetic sources of variation (*Pop*, *Fam*; Table 7 and 8). Most of the variation in photochemical parameters was partitioned by these factors or was caused by family plastic responses to light, water and/or temperature stresses (*GxE* interactions, Table 7). This finding is consistent with Baquedano et al. [30]. The authors found that phenotypic plasticity blurred the ecotypic divergence of fluorescence traits, which could hide differences among *P. halepensis* populations and maintain genetic variation unavailable to selection.

**Table 7:** Percentages of variance (%).

Sources of variation	*F*_v_/*F*_m_	Φ_PSII_	*qP*	*NPQ*
***Site***	0	0	0	5
***Winter***	4	30	38	14
***Site*** ** x ** ***winter***	49	2	4	3
***Pop***	0	0	0	0
***Pop*** ** x ** ***site***	0	0	0	0
***Pop*** ** x ** ***winter***	0	0	0	0
***Pop*** ** x (** ***site*** ** x ** ***winter*** **)**	0	0	0	0
***Fam*** ** (** ***Pop*** **)**	0	0	0	0
***Fam*** ** (** ***Pop*** **) x ** ***site***	1	0	5	0
***Fam*** ** (** ***Pop*** **) x ** ***winter***	0	2	3	0
***Fam*** ** (** ***Pop*** **) x (** ***site*** ** x ** ***winter*** **)**	1	8	9	11
***Error***	45	57	41	66

Abbreviations are the same as those in Table 2. Empty boxes denote zero values.

**Table 8:** Percentages of variance by *site* and *winter* (%).

		Cold winter		Warm winter	
Parameter	Sources of variation	Site *XE*	Site *ME*	Site *XE*	Site *ME*
***F*** **_v_/** ***F*** **_m_**	***Pop***	0	0	0	1
***F*** **_v_/** ***F*** **_m_**	***Fam*** ** (** ***Pop*** **)**	1	3	8	2
***F*** **_v_/** ***F*** **_m_**	**residual**	84	12	44	45
**Φ_PSII_**	***Pop***	0	0	0	0
**Φ_PSII_**	***Fam*** ** (** ***Pop*** **)**	12	7	12	9
**Φ_PSII_**	**residual**	42	40	52	26
***qP***	***Pop***	0	0	3	0
***qP***	***Fam*** ** (** ***Pop*** **)**	11	2	17	25
***qP***	**residual**	57	30	30	25
***NPQ***	***Pop***	4	0	3	0
***NPQ***	***Fam*** ** (** ***Pop*** **)**	7	5	6	7
***NPQ***	**residual**	41	43	45	40

Abbreviations are the same as those in Table 2.

The sensitivity of *P. pinaster* to low temperatures supports previous findings regarding Mediterranean species, e.g., low winter temperatures had a greater impact on *F_v_*/*F_m_* than high temperatures or drought during the summer in *Quercus suber* populations [31]. Moreover, when low winter temperatures were associated with low soil moisture, Mediterranean species showed the lowest values of *F_v_*/*F_m_* [32] and chronic photoinhibition [28]. Subtropical [33] and temperate [34] species have also displayed the lowest values of the maximum (*F_v_*/*F_m_*) and effective (Φ_PSII_) quantum yield of PSII during winter.

### Genetic variation in PSII photochemical parameters in response to winter stress and freezing temperatures (LT_50_)

Genetic variation in *P. pinaster* photochemical parameters due to winter stress was found mostly at the family level (Table 8). There was greater intra-population variation than inter-population variation. Population variation was displayed exclusively at the xeric site. This finding is in line with a previous study [3] and suggests that population differentiation [35] and population selection [30] take place under adverse conditions. Measurements under favorable growing conditions in common garden experiments overcome the problem of confounding environmental effects with genotypic differences. However, some differences in physiological traits among genotypes that reflect adaptation to their climate of origin may be appreciated only when plants are exposed to stress. Population differences at the xeric site could be related to the higher cumulative radiation, lower precipitation and more extreme temperatures of the site (Fig. 2). This is consistent with the results of Colom et al. [36], who observed small but significant differences between populations in *F_v_*/*F_m_* and effective PSII quantum yield (Φ_PSII_) at increasing light intensities. Aranda et al. [31] also found differences among populations of *Quercus suber* during periods of low winter temperatures, when greater reductions in *F_v_*/*F_m_* (0.2–0.3) caused the highest population variance.

The low variation in the photochemical parameters attributed to the population effects found in this experiment is in agreement with Lopez et al. [37] and Baquedano et al. [30], who observed that photochemistry did not vary among populations in the control or drought treatments of *Pinus canariensis* and *Pinus halepensis*, respectively. Nevertheless, the small but highly significant population differences in photochemical performance that were expressed at the *XE* were related to the climatic origin of the seed source. The population from France, *Mimizan*, which experience mild winters due to the Atlantic influence, presented significantly higher values of Φ_PSII_ and *qP* during the warm winter. Photoprotection mechanisms, such as a high efficiency of heat dissipation, may have allowed *Mimizan* to reach higher Φ_PSII_ and *qP* values after the warm winter but not after the cold winter, when there were sustained decreases in *F_v_*/*F_m_*, Φ_PSII_ and *qP*. The *Arenas* population, originally from a location in interior and continental Spain with extreme maximum and minimum temperatures, displayed lower thermal energy dissipation (*NPQ*) in both winters.

Our results suggest that the LT_50_ of needles and stems, estimated by *F_v_*/*F_m_*, can be used to test the freezing tolerance of *P. pinaster* intraspecifically. Differences among populations in needle LT_50_ were consistent with the minimum temperatures of their climate of origin. This is in accordance with Climent et al. [38], who found that the LT_50_, estimated by the electrolyte leakage method, was highly correlated with the mean temperature of the coldest month in Mediterranean pine species of contrasting thermal habitats. We observed less freezing damage in the *Tamrabta* and *Arenas* populations, which are from continental climates, than in *Mimizan* and *Oria*, which are from coastal climates, reflecting adaptation to the ecological niches provided by the original climates. These results are consistent with similar studies in other coastal and interior conifer populations [26]. Even though the populations do not experience these minima temperatures at their sites of origin, this is useful information for possible reforestations in northern and colder latitudes in the future.

The LT_50_ of the cortical bark chlorenchyma was more variable (higher standard errors) than needle LT_50_. Plant material subjected to a range of freezing temperatures either escapes damage or is completely killed, and only a narrow range of temperatures induce partial damage. Taking into account the results of this research, we would expect that more accurate values would be obtained in future experiments that study several freezing temperatures in smaller increments (1–2°C) within the critical range of temperatures (from −20°C to −30°C for needles and from −30°C to −40°C for stems).

The maritime pine, as a Mediterranean conifer, must survive fluctuations in temperature, water soil availability, vapor deficits and high light irradiance during the year. The regulation of the electron transport processes by both environmental and genetic mechanisms is an advantage for the species. This study documents genetic variation in fluorescence traits, both between and within populations, but mostly within populations. However, variation due to environmental conditions accounted for the major proportion of the total variance. Although we found highly significant differences between families, most of the genetic variation was due to the interaction between *family x environment* (*site*, *winter*, *site x winter*). Further research is needed before considering intra-specific selection for photochemical efficiency in *P. pinaster*, and future studies should be directed toward finding differences among families. The investigation of a large number of genotypes over a wide range of environments in field tests would be necessary to understand the genes that regulate photosynthetic processes that can be monitored by chlorophyll fluorescence and their effects on survival and growth. The identification of quantitative relationships between abiotic stresses and photochemical activity could be useful to assess the plasticity of families and populations and for developing selection criteria for abiotic stress tolerance. In this work, we found that there is a potential for the selection of more frost tolerant *Pinus pinaster* populations, which is an advantage for a species that have to survive in unpredictable environments, especially in continental areas or at high altitudes.

### Conclusion

We provide evidence for *P. pinaster* sensitivity to winter stress and for intraspecific variation in the PSII photochemical parameters in response to winter stress, mainly at the family level.

LT_50_ obtained by *F_v_/F_m_* was consistent with the thermal ecological niche of the populations and can be reliably used to find differences in the frost tolerance of needles and stems among *P. pinaster* populations.

## Materials and Methods

### Ethics Statement

All necessary permits were obtained for the described field studies. Permissions required for field studies were obtained from the Environmental Departments of the Autonomous Governments of Aragon and Galicia.

### Study site and plant material

The range of *Pinus pinaster* (Ait.) extends over the occidental Mediterranean basin and the southern European Atlantic coast of France and Spain. This relatively small area covers a wide range of climates, from arid to humid conditions, and altitudes from sea level up to 2000 m. It is a species widely used in Spanish reforestation programs and for tree breeding. Its highly fragmented distribution is explained by the discontinuity and high altitudes of the mountain ranges in southwestern Europe, which led to the isolation of geographically close populations and to several adaptations for growth and survival in distinct climates [39].

Open-pollinated siblings (individuals with one parent in common and the other parent unknown) were collected in natural stands of maritime pine in France, Spain and Morocco, and the seedlings were grown in nurseries. Progeny trials were established throughout Spain. We chose two progeny trials, located at Parderrubias, NW Spain (mesic site, *ME*) and Calcena, NE Spain (xeric site, *XE*). Sites were chosen to compare tree behavior under contrasting temperature and precipitation regimes. The *ME* is situated near the Atlantic Ocean, with a wetter and milder climate than the *XE*, which is continental with lower winter temperatures. Both sites undergo drought during the summer. Climatic data were obtained from the meteorological stations of Allariz, approximately 14 km from the *ME* (42°11′N, −7°48′W, 476 m a.s.l.), and Aranda de Moncayo, approximately 24 km from the *XE* (41°35′N, −1°47′W, 827 m a.s.l.).

At the *ME*, two-year-old seedlings were planted in 2005, separated from each other by 2×3 m, in a randomized complete block design with 4 replications of 71 blocks, 225 families and 4 plants per experimental unit (a total of 16 plants per family). At the *XE*, one-year-old seedlings were planted in 2004, separated from each other by 2×3 m, in an α-lattice incomplete block design with 3 replications of 65 blocks, 8 families per block and 4 plants per experimental unit in a nested structure (families within populations). The plots were blocked by their position on the slope.

We selected three populations from each of the trial sites (*Arenas*, central Spain; *Oria*, southeast Spain; *Mimizan*, southwest coast of France) to test the photochemical parameters during the winters of 2006 and 2007. For the freeze test, we added the Moroccan *Tamrabta* population and measured 4 populations from the continental site (*XE*) exclusively. Location and climatic data for the trial sites and populations are presented in Figure 2 and Table 1.

### Winter stress

At *XE*, physiological measurements were performed, contingent on the availability of plant material. Weather conditions limited the number of measurements at the *ME*. We collected data from 3 populations, evaluating 6 to 10 families per population and 4 to 8 trees per family. On each day of measurements, families and populations were selected at random in the field.

On clear and sunny winter days, photosynthetically active radiation (PAR) was 2198±23 µmol photons m^−2^ s^−1^ at the *XE* and 2099±17 µmol photons m^−2^ s^−1^ at the *ME*. Chlorophyll fluorescence was measured between 10:00 a.m. and 1:00 p.m. (13:00, solar time) for three consecutive days at the *XE*, followed by another three consecutive days at the *ME*, with a modulated portable fluorescence monitoring system (Hansatech Instruments, FMS 2). Needles were illuminated with light from the FMS 2. PAR was adjusted to 1500 µmol photons m^−2^ s^−1^ at the *XE* and 1360 µmol photons m^−2^ s^−1^ at the *ME* to avoid photoinhibition. A portable computer was coupled to the FMS 2 to record any changes in the kinetics of the modulated chlorophyll fluorescence.

The photochemical parameters, Φ*_PSII_*, *F_v_/F_m_* and *q_P_*, and the photoprotective mechanism *NPQ*, provide valuable information about the electron transport system and the conversion and dissipation of the excess excitation energy into heat and can be used as indicators of plant stress [14]. Φ*_PSII_* and *F_v_/F_m_* reflect the actual and maximum potential efficiency of the excitation energy captured by open PSII centers, respectively. While Φ*_PSII_* indicates the proportion of absorbed energy that is used in photochemistry, the photochemical quenching, *q_P_*, indicates the proportion of PSII reaction centers that are open and shows the level of fluorescence quenching due to the rapidly relaxing redox state of the primary quinone of PSII, Q_A_. In contrast, the non-photochemical quenching, *NPQ*, shows the level of fluorescence quenching due to the slowly relaxing high energy status of the thylakoids, i.e., the ΔpH-dependent processes that lower the efficiency of PSII as the rates of electron transport and carbon metabolism reach saturation at high photon fluxes. NPQ is directly proportional to the rate constant for the energy dissipation as heat.

The experimental protocol was essentially as that described by Genty et al. [40], with some modifications [41]. Several needles from the current year were dark-adapted using leaf clips provided with the FMS 2 and were kept in darkness for 30 min before estimating the minimum (*F_o_*) and maximum (*F_m_*) chlorophyll fluorescence at predawn. *F_o_* was measured by switching on the modulated light at 0.6 kHz. *F_m_* and *F′_m_* were measured at 20 kHz with a pulse of 6000 µmol photons m^−2^ s^−1^ of white light for 1 s. *F_o_* and *F′_o_* were measured in the presence of far-red light (7 µmol photons m^−2^ s^−1^) to completely oxidize the PSII acceptor side [41]. The actual fluorescence level (*F_s_*) was obtained when fluorescence reached a steady-state value, which was verified visually on the computer screen. After the saturating pulse of light for determining the maximum fluorescence in the light (*F′_m_*), the fluorescence signal was allowed to re-equilibrate. When the signal reached the same steady-state level observed prior to the saturating pulse of light, it was monitored for approximately 20 s to attain the *F′_o_* level. The maximal photochemical efficiency of PSII, *F_v_*/*F_m_*, measured in the dark-adapted leaves, was given by the equation (*F_m_*−*F_o_*)/*F_m_* [42]. Actual PSII efficiency (Φ_PSII_) was estimated as (*F′_m_*−*F_s_*)/*F′_m_* [43]. Photochemical quenching (*qP*) was estimated as (*F′_m_*−*F_s_*)/*F′_v_* [42]. Non-photochemical quenching (*NPQ*) was estimated as (*F_m_*−*F′_m_*)−1 according to Bilger and Björkman [44].

### Water potential

Predawn water potential, an indicator of plant water status at the moment of measurement, was obtained with a Scholander pressure chamber. Shoots from the outer parts of the crowns of ten individuals were taken from trees on the upper, middle and lower part of the slope. Shoots were wrapped in plastic film, cut with pruning shears and measured immediately.

### Freeze test

At the *XE*, branches from ten individuals per population were randomly selected in the field for each freezing temperature. We used current-year needles and branches from the three populations tested for winter stress and also added a new population, *Tamrabta*, to cover a wider range of winter temperatures and altitudes from the provenances. Immediately after collection, the samples were stored in a black bag inside a thermoelectric refrigerator at 4°C and brought to the laboratory for measurements. The study was conducted during February 2007.

We adapted a commercial freezer to obtain the temperature profiles. Cooling rates were established through an industrial controller (PMA Prozess-und Maschinen-Automation GmbH Mod. KS90, Germany) acting on a heating block with forced convection. Freezing was provided by the continuous performance of the freezer engine. Thermal homogeneity was achieved through a microfan system inside the chamber. This device provided a thermal stability of ±0.1°C onset temperature along the whole thermal profile. The samples (10 twigs per test temperature and population) were exposed to various freezing temperatures between 0 and −25°C. The rate of cooling was 5°C h^−1^, and the samples were exposed to the freezing temperature for 1 h. This device was not able to achieve temperatures below −25°C, so temperature profiles between −30°C and −60°C were generated with a commercial freezer (CHF 250/80, Ingenieria de Climas, Barcelona, Spain) equipped with a PID digital temperature controller. The same experimental procedure described above was used for the exposition time and rate of cooling. The control temperature was 4°C. The freezing tolerance of the needles and stems (cortical bark chlorenchyma) was assessed by means of chlorophyll fluorescence. Curves were fitted to a logistic sigmoid function by regression analysis. LT_50_ was estimated as the inflection point of the sigmoid function:
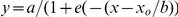
where 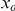 is the exposure temperature, *y* is the value of the variable used for the estimation of freezing tolerance, *a* defines the asymptote of the function, and *b* is the slope at the inflection point 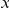.

### Statistical analysis

To characterize the environments of the populations and testing sites, we constructed a dummy index based on climatic data, including as much variability as possible. Using a GIS (Geographic Information System), we extracted values for the 19 bioclimatic variables provided by the “Worldclim” model [45]. For each variable, we calculated the mean value for a circular surface with a 10 km radius centered on the coordinates of the testing sites and population provenances. The climatic index used for comparison purposes was the first component value obtained from a principal component analysis (PCA) of these bioclimatic data.

According to the experimental design, and on the basis of the subsampling that was performed, a set of mixed models was used for all variables. The normality and homoscedasticity of the data were confirmed.

The general model established was as follows:
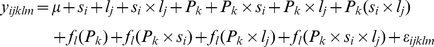
where

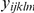
 is the value of the variable for the *m*_th_ seedling from the *k*_th_ population within the *l*_th_ family measured at the *j*_th_ *site* during the *i*_th_ winter;

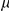
 is the overall mean of the variable;
*s_i_* is the effect of the *i*_th_ winter (*i* = 1–2);
*l_j_* is the effect of the *j*_th_ site (*j* = 1–2)
*P_k_* is the effect of the *k*_th_ population (*k* = 1–3);
*f_l_* is the effect of the *l*_th_ family (*l* = 1–10) within the *k*_th_ population;

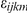
 is the residual (*m* = 1–4).

This model was applied both generally and individually, by winter and site, removing the sources of variation, “*site*” and “*winter*”, from the model. The model was analyzed as a mixed model with fixed (*site*, *winter*, *population* and *family*) and random (*error*) effects, and the components of variance were obtained by restricted maximum likelihood (REML). The best linear unbiased estimators and predictors (BLUE and BLUP) for fixed and random factors, respectively, were obtained using SAS [46].

Pearson correlation coefficients were obtained to analyze the relationships between the variables considered.
